# Radiation vs surgery for early-stage laryngeal verrucous carcinoma: A population-based propensity score matched-study

**DOI:** 10.1371/journal.pone.0275271

**Published:** 2022-10-31

**Authors:** Yukinori Takenaka, Atsuhiko Uno, Hidenori Tanaka, Norihiko Takemoto, Kengo Nozaki, Hidenori Inohara

**Affiliations:** 1 Department of Otorhinolaryngology-Head and Neck Surgery, Osaka University Graduate School of Medicine, Suita, Osaka, Japan; 2 Department of Otorhinolaryngology, Osaka Police Hospital, Osaka, Japan; 3 Department of Otorhinolaryngology-Head and Neck Surgery, Osaka General Medical Center, Osaka, Japan; University Hospital Zurich, SWITZERLAND

## Abstract

**Background:**

Verrucous carcinoma (VC) is a rare variant of squamous cell carcinoma. Although VC is considered radioresistant, concrete evidence for this is absent.

**Methods:**

We obtained data on VC treated with surgery or radiation from the Surveillance, Epidemiology, and End Results database. Treatment selection bias was reduced by propensity score matching. Overall survival (OS) and disease-specific survival (DSS) rates were estimated using the Kaplan-Meier method. Hazard ratios (HRs) were estimated using Cox proportional hazards models.

**Results:**

Five-year OS rates in the radiation and surgery groups were 72.7% and 72.0%, respectively (*P* = 0.111); five-year DSS rates in the same were 86.7% and 88.4%, respectively (*P* = 0.234). HRs of radiation compared with surgery were 1.68 (95% confidence interval (CI), 0.96–2.95) for OS and 1.95 (95% CI, 0.69–5.53) for DSS.

**Conclusions:**

Similar prognoses were observed in patients with VC treated with radiation and surgery. VC can be treated using radiation.

## Introduction

Verrucous carcinoma (VC) is a well-differentiated variant of squamous cell carcinoma (SCC) that was first reported by Ackerman et al. in 1948 [1]. The larynx is the second most common site for VC in the upper aerodigestive tract, following the oral cavity [2]. Laryngeal VC accounts for 0.9% of laryngeal malignancies [3] and 1.25% of early-stage laryngeal SCC cases. Early-stage laryngeal SCC is usually treated with radiotherapy (RT) or surgical monotherapy. However, VC tends to be treated with surgery rather than RT. One reason for this is the possibility of anaplastic transformation caused by RT, which was reported in early studies [4,5]. Another reason is the radioresistant nature of VC [4]. Thus, RT for VC should be avoided, whenever possible.

In 1993, Hagen et al. reported a local failure rate of 51% and anaplastic transformation rate of 11% in 37 patients with laryngeal VC treated with RT [4]. In contrast, Huang et al. (2009) found no cases of anaplastic transformation following RT in a retrospective case series of 62 patients [6]. Thus, recent studies have negated the possibility of radiation-induced anaplastic transformation despite earlier studies. However, the radioresistance of VC has not yet been properly assessed. A population-based study by Dubal [3] demonstrated a statistically significant difference in disease-specific survival (DSS) among different treatment modalities in patients with laryngeal VC. However, stratification or adjustment for possible confounding factors was not performed.

A randomized controlled trial is the best method for comparing treatment results between different treatment modalities. However, conducting such trials for laryngeal VC is almost impossible because of its rarity. The propensity score matching method has been increasingly used in quasi-experimental designs. This method can control for known confounding factors and compare the results of different treatments in cohort studies. In the present study, we collected data on laryngeal VC from a population-based database and investigated the prognosis of patients with laryngeal VC according to the treatment modality. Our study aimed to investigate whether RT could be the treatment of choice for early-stage laryngeal VC.

## Patients and methods

Approval from the institutional review board of Osaka University Hospital was waived because the analyzed data were publicly available and kept anonymous.

### Data retrieval

Individual patient data were retrieved from the Surveillance, Epidemiology, and End Results (SEER) Research Plus Data, 18 registries, and November 2020 Sub using SEER Stat software, version 8. 3. 9. 2 (National Cancer Institute, Bethesda, MD, USA). The inclusion criteria were as follows: (1) laryngeal cancer (Site recode ICD-O-3/WHO 20008, “larynx”) (2) histologically confirmed VC (ICD-O-3 Hist/behav, 8051/3) (3) stage I or II according to the American Joint Committee on Cancer (AJCC) staging system, sixth edition (stage for cases diagnosed between 2000 and 2003 was determined using EOD 10 code), (4) types of reporting source were ’Hospital inpatient/outpatient or clinic’, ’Radiation treatment or medical oncology center (2006+)’, ’Laboratory only (hospital or private)’, ’Physician’s office/private medical practitioner (LMD)’, ’Nursing/convalescent home/hospice’, ’Other hospital outpatient unit or surgery center (2006+)’, (5) treated with external RT (coded as “beam radiation”) or surgery (surgery code, 10–90) monotherapy, and (6) diagnosed between 2000 and 2015.

The exclusion criteria were (1) patients receiving chemotherapy and (2) subsites coded as “larynx, NOS” or “overlapping lesions”.

### Statistical analysis

One-to-two propensity score matching was carried out using the nearest matching method with a caliper of 0.20. Covariates for matching were age, marital status, race, sex, stage, subsite, and year of diagnosis. Matching one RT case to two surgery cases was used to increase precision [7].

After matching, patient characteristics were compared using the chi-square test for the association between categorical variables and the Kruskal-Wallis test for the associations between categorical and continuous variables. DSS and overall survival (OS) rates were estimated using the Kaplan-Meier method and subsequently compared using the log-rank test. Multivariate analyses were performed using a Cox proportional hazards model. The cumulative incidence of death was calculated using non-parametric cumulative incidence functions and compared using Gray’s test. Statistical significance was set at *P* < 0.05. All statistical analyses were performed using EZR [8] (Saitama Medical Center, Jichi Medical University, Saitama, Japan), a graphical user interface for R (R Foundation for Statistical Computing, Vienna, Austria).

The primary endpoint was overall survival. The secondary endpoints were disease-specific survival and the cumulative incidence of death due to other causes.

## Results

### Patient characteristics

The SEER database contains 162 cases of VC that met the inclusion criteria. After excluding 14 cases that met the exclusion criteria, 148 patients were included. The number of RT and surgery cases was 38 and 110, respectively. Propensity score matching yielded 33 patients treated with RT and 66 treated with surgery (Table 1). There were no significant differences in patient characteristics between the two groups. During the follow-up period, 15, 36, and 2 patients died from disease, other causes, and unknown causes, respectively. The median follow-up period of surviving patients was 136 months.

**Table 1:** Patient characteristics.

	Radiation (N = 33)		Surgery (N = 66)		*P* value	SMD
	No.	%	No.	%		
Age Median Range	62 44–84		62 28–90		0.905	0.01
Sex Male Female Marital status Married Single Divorced/separated/widowed Unknown Race White Other Subsite Glottis Supraglottis Stage I II Year of diagnosis 2000–2005 2006–2010 2011–2015	32 1 20 5 4 4 29 4 32 1 24 9 17 10 6	97 3 61 15 12 12 88 12 97 3 73 27 52 30 18	66 0 37 14 7 8 59 7 63 3 51 15 28 22 16	100 0 56 21 11 12 89 11 96 4 77 23 42 33 24	0.333 0.922 1.000 1.000 0.627 0.844	0.25 0.161 0.048 0.079 0.105 0.060

Abbreviations: SMD, standardized mean difference.

### Overall survival

**Fig 1:**
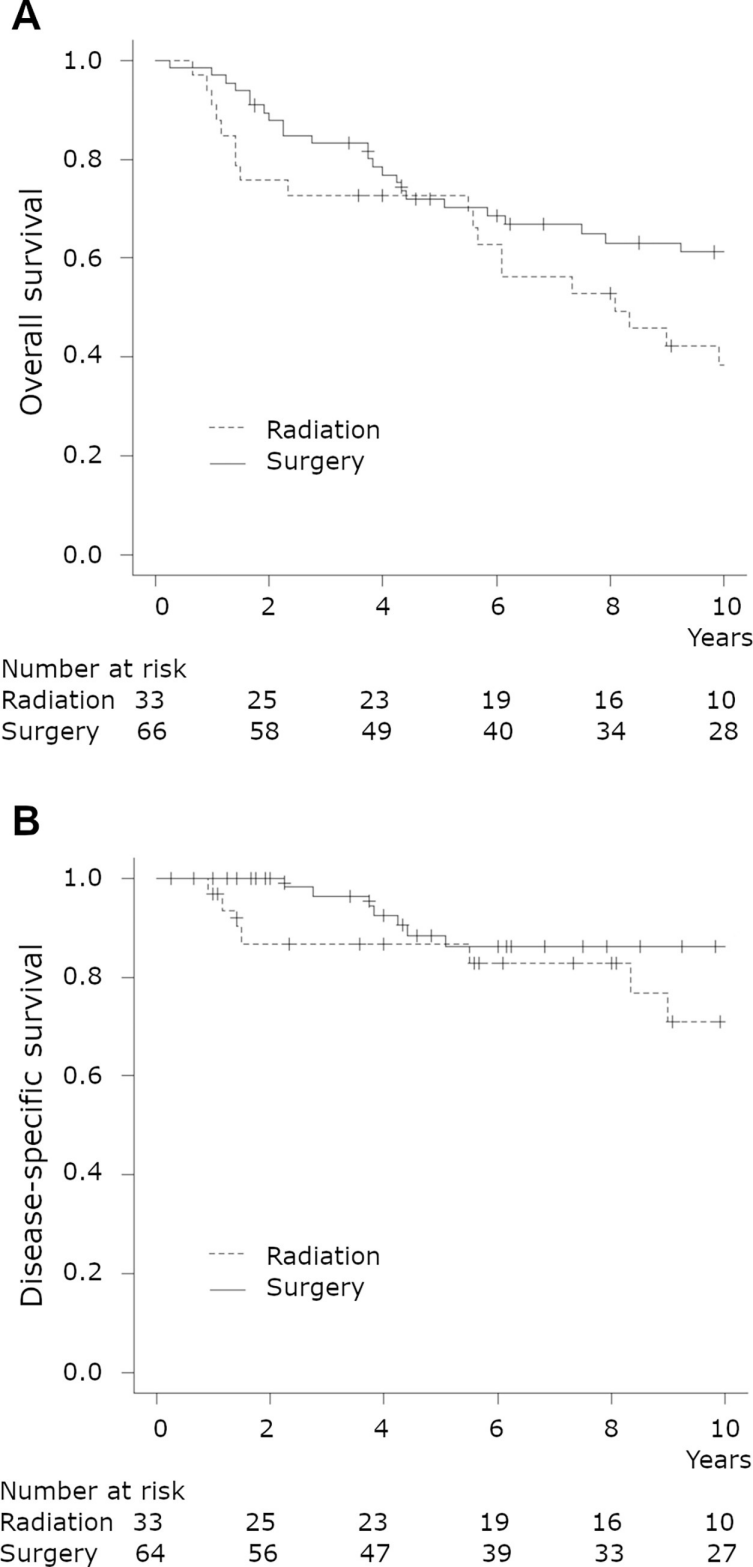
Kaplan-Meier curves according to treatment modalities. A, overall survival. B, disease-specific survival.

Fig 1A shows the OS according to the treatment modality. The five-year OS rates were 72.7% (95% confidence interval (CI) 54.1–84.8)) for the RT group and 72.0% (95% CI 59.3–81.4)) for the surgery group (*P* = 0.111). Table 2 shows the unadjusted and adjusted hazard ratios (HRs) for OS. The treatment modality was not a prognostic factor for OS. The adjusted HR for RT compared with surgery was 1.68 (95% CI, 0.96–2.95). Age was the only independent prognostic factor for OS (HR 1.07 (95% CI, 1.04–1.09)).

**Table 2:** Cox proportional hazard model for overall survival.

	Univariate analysis			Multivariate analysis		
Characteristics	HR	95% CI	*P* value	HR	95% CI	*P* value
Therapy Surgery Radiation Age Stage I II	Ref 1.55 1.06 Ref 0.83	0.90–2.68 1.01–1.09 0.42–1.66	0.116 <0.001 0.603	Ref 1.68 1.07 Ref 0.95	0.96–2.95 1.04–1.09 0.48–1.92	0.072 <0.001 0.897

Abbreviation: CI, confidence interval, HR, hazard ratio.

### Disease-specific survival

Fig 1B shows the DSS according to the treatment modality. The five-year DSS rates were 86.7% (95% CI, 68.3–94.8)) for the RT group and 88.4% (95% CI 75.9–94.6)) for the surgery group (*P* = 0.234). Table 3 shows the unadjusted and adjusted HRs for DSS. Treatment modality did not affect DSS. The adjusted HR for RT compared with surgery was 1.95 (95% CI, 0.69–5.53). Age was the only independent prognostic factor for DSS (HR 1.05 (95% CI, 1.01–1.11)).

**Table 3:** Cox proportional hazard model for disease-specific survival.

	Univariate analysis			Multivariate analysis		
Characteristics	HR	95% CI	*P* value	HR	95% CI	*P* value
Therapy Surgery Radiation Age Stage I II	Ref 1.83 1.05 Ref 0.86	0.66–5.06 1.01–1.10 0.24–3.05	0.242 0.025 0.816	Ref 1.95 1.05 Ref 1.05	0.69–5.53 1.01–1.11 0.29–3.82	0.208 0.017 0.940

Abbreviation: CI, confidence interval, HR, hazard ratio.

### Cumulative incidence of death

**Fig 2:**
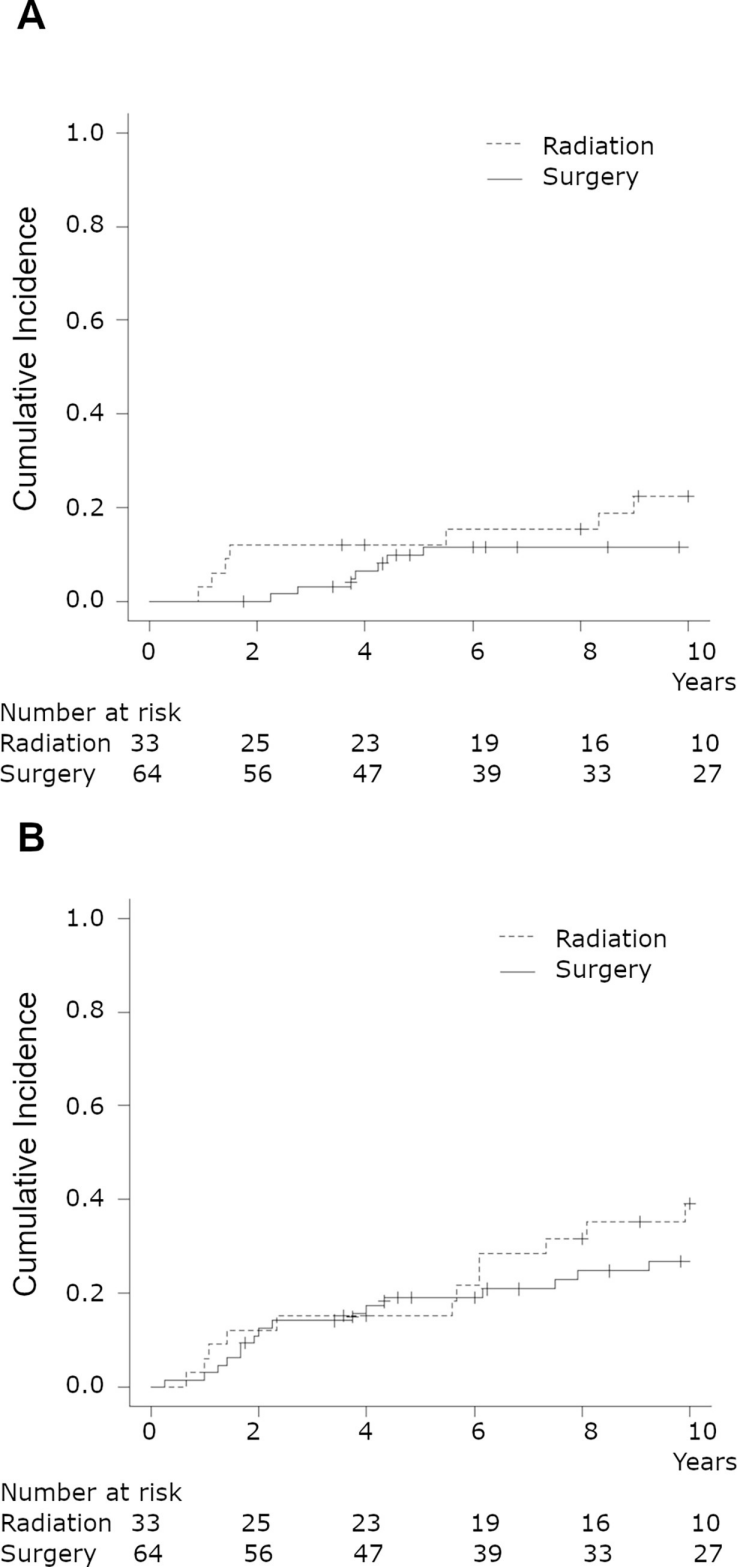
Cumulative incidence for death according to treatment modalities. A, death due to cancer. B, death due to other cause.

The cumulative incidence of death due to VC and other causes is shown in Fig 2A and 2B, respectively. The mortality rates due to VC at five years were 12.1% in the RT group and 9.8% in the surgery group (*P* = 0.285). The mortality rates due to other causes were 15.2% and 19.1% in the RT and surgery groups, respectively (*P* = 0.391).

## Discussion

In this study, we compared the results of RT and surgery in patients with laryngeal VC using a quasi-experimental design. We demonstrated that the prognosis was comparable between the two modalities.

Several rare variants of SCC arise in the upper aerodigestive tract, including VC, lymphoepithelial carcinoma, papillary, spindle, and adenosquamous SCCs [9]. These variants have different prognoses and sensitivities to specific treatments for conventional SCC. VC and papillary SCC have a better prognosis than other laryngeal malignancies [10,11], whereas adenosquamous SCC has a worse prognosis [12]. In addition, radiosensitivity varies among SCC variants. VC is considered radioresistant [5], while lymphoepithelial carcinoma shows a good response to RT [9]. Therefore, surgery is preferred to RT for laryngeal VC. A recent population-based study showed that 18.2% of patients with early-stage laryngeal VC were treated with RT alone, in contrast to 49.9% of patients with conventional SCC. Despite the predilection for surgery, some patients undergo RT because of intolerance to general anesthesia, aversion to surgery, and low voice quality after surgery. Huang et al. reported a case series of 62 patients with laryngeal VC treated with RT [6]. No anaplastic transformation was observed during the median follow-up period of 11 years. The local control rate, excluding the effect of salvage surgery, in all stages of VC was 66% at five years, and the larynx was preserved in 81% of patients when salvage larynx preservation surgery was included. Their study included advanced-stage disease and older patients treated with outdated RT methods. The present study investigated only patients with early-stage disease who were diagnosed after 2000. We found no significant difference in survival between patients treated with RT and those treated surgically. The five-year DSS for patients with laryngeal VC treated with RT was 86.7%, similar to 83.9% in patients with early-stage laryngeal conventional SCC treated with RT [13]. Therefore, RT may be an option for early-stage laryngeal VC.

Our study has several strengths. First, using a population-based database, we analyzed 99 cases of early-stage laryngeal VC over a 15-year period. Considering the rarity of VC, such a number of cases could not be collected with from a single institute. Second, we used propensity score-matching. We were able to control for several confounding factors that could affect treatment selection. Finally, we conducted competing mortality analysis. Surgically treated patients may experience postoperative complications; aspiration can be a problem after larynx-preserving surgery; radiation can also cause secondary primary malignancies. These conditions often lead to non-laryngeal cancer death. Thus, a comparison of other causes of death should be considered when selecting treatment.

The limitations of this study are as follows. First, SEER data do not contain information that can affect prognosis and treatment decisions. The major etiologic causes of laryngeal cancer are habitual drinking and smoking, which can cause comorbidities including secondary primary malignancies, heart disease, and pulmonary diseases. Patients with severe pulmonary disease may not tolerate general anesthesia. Patients with other malignancies may undergo simultaneous surgeries. Therefore, these comorbidities significantly influenced the selection between RT and surgery. However, data on comorbidities were lacking, resulting in insufficient control of the confounding factors. The location and extent of the tumor also affect treatment decisions. SEER data provides tumor subsites as supraglottis, glottis, and subglottis, and neither offers more detail on the location nor extent of the tumor. Because of the lack of this information, we could not offer recommendations regarding which patients are suitable for RT. Second, details of RT, including total dose, duration, and methods, are not elaborated in the database. Recent technical advances in RT, specifically hyperfractionation and accelerated fractionation, have improved local control of laryngeal cancer [14]. Well-differentiated tumors benefit more from reducing the overall treatment time than their dedifferentiated counterparts [15]. Therefore, VC, a well-differentiated variant of SCC, is a promising candidate for accelerated fractionation. If we analyze only cases treated with accelerated faction, the prognosis would be better than that in the present study. Finally, we only evaluated survival. Local control failure of RT can be salvaged with surgery. In a case series of laryngeal VC treated with RT, local recurrence was observed in 26% of T1 and 31% of T2 cases, respectively [6]. Local recurrence has been reported to be successfully treated in most cases. Therefore, a good prognosis in patients treated with RT may be spurious because of salvage surgery.

In conclusion, we demonstrated that laryngeal VC patients treated with RT had a prognosis similar to that of surgically treated cases. Therefore, RT can be considered as an option for early-stage laryngeal VC. Whether RT is the primary choice of treatment has not yet been elucidated. Given the rarity of laryngeal VC, prospective studies are not feasible. Therefore, well-designed multicenter retrospective studies are required.

## Supporting information

S1 Data (XLSX)

S2 Data (XLSX)
